# Supporting the spirituality of older people living with dementia in nursing care: A hermeneutic phenomenological inquiry into older people's and their family members' experiences

**DOI:** 10.1111/opn.12514

**Published:** 2022-11-15

**Authors:** Kristiina Toivonen, Andreas Charalambous, Riitta Suhonen

**Affiliations:** ^1^ Department of Nursing Science University of Turku Turku Finland; ^2^ Department of Nursing Science Cyprus University of Technology Limassol Cyprus; ^3^ Welfare Services Division Turku University Hospital & City of Turku Turku Finland

**Keywords:** dementia, hermeneutic phenomenology, older people nursing, qualitative study, Ricoeur, spirituality, supporting spirituality

## Abstract

**Background:**

Supporting spirituality is an essential aspect of the holistic nursing care of older people living with dementia. Spirituality is defined as a search for answers to questions about the meaning and purpose of life and the individual's relationship with the sacred or transcendent. This relationship may or may not involve an affiliation with a specific religion.

**Objective:**

To understand how older people living with dementia and their family members experience spirituality and its support in nursing care.

**Design:**

A qualitative study informed by the principles of Ricoeurian hermeneutic phenomenology.

**Settings:**

We conducted the study in home care and long‐term care settings in Southern Finland.

**Participants:**

We collected data between 2017–2020 from a purposive sample of 10 older people living with dementia and their 9 family members (*n* = 19).

**Methods:**

We used interviews to collect data and adapted and used Ricoeur's theory of interpretation as a method for analysis.

**Results:**

The findings of this study show that older people living with dementia need spiritual support in nursing care based on their personal understanding of spirituality. The four elements of this spirituality that emerged were: religion, meaningful relationships, nature, and art. The participants addressed some challenges to spiritual support in the nursing care of older people living with dementia including: the competence and abilities of nursing, time available, presence and experience.

**Conclusions:**

Older people living with dementia and their family members consider spiritual support an important aspect of nursing care. To support the spirituality of these older people, the elements of spirituality need to be understood as these are central to each person's spiritual position. Additionally, spiritual support requires understanding knowledge, experience, time and presence, to manage all four elements with individuals.

**Summary statement of implications for practice**

**What does this research add to existing knowledge in gerontology?**

- Limited research has been undertaken focusing on supporting the spirituality of older people living with dementia.
- Older people living with dementia have rarely been informants in qualitative studies focusing on spiritual support.
- There are different ways through which the spirituality of older people living with dementia can be supported.
- An understanding of the personal sense of spirituality of each older person living with dementia is central to their support framework. Currently, some nurses may not have the time, ability, understanding or presence to deliver spiritual care optimally.

**What are the implications of this new knowledge for nursing care with older people?**

- The spiritual needs of older people living with dementia are personalised and should be addressed as such in nursing care.
- With better education and experience nursing professionals, as a whole, could improve the spiritual care they provide to older people living with dementia.

**How could the findings be used to influence policy, practice, research, or education?**

- Nurses could learn to identify the spiritual needs of older people living with dementia more comprehensively and respond to those needs positively in practical nursing.
- More research on this topic could focus on the role of the environment in supporting the spirituality of older people living with dementia. Tools for assessing the spiritual needs of older people living with dementia are needed.

## 1 INTRODUCTION

Even in a secular society, spiritual needs are important (Timmins et al., 2016), and seem to increase with age (Moberg, 2012, p. 85). Supporting spiritual needs is therefore, an important part of quality care for older people (Cleland et al., 2021). The experience of spirituality is subjective, so individuality is central to this support (Toivonen et al., 2015).

Around 50 million people live with dementia worldwide (WHO, 2017) and this population is expected to double every 20 years (Prince et al., 2015). The costs of providing quality care for people with dementia are also increasing (Wittenberg et al., 2020) especially as independence reduces. Dementia causes disability and dependency among older people all over the world (World Health Organization, 2012). The World Health Organisation's (WHO) “Global action plan on the public health response to dementia 2017–2015” states that people living with dementia should be able to live valuable and meaningful lives (WHO, 2017).

The personal significance of spirituality does not disappear with the onset of dementia and may even become more important (Daly et al., 2019; Scott, 2016). Spirituality can help older people with dementia to reduce stress and anxiety and increase the experience of meaning in life and hope (Scott, 2016). Older people living with advanced dementia may depend on other people to provide support to meet their spiritual needs (Toivonen et al., 2015). There is a need to strengthen and develop the role of nurses to manage this support (Timmins et al., 2016) as part of everyday nursing practice requiring little financial investment (Toivonen et al., 2015).

The concept of spirituality within nursing has been analysed and is well reported (e.g. Il et al., 2017; Murgia et al., 2020; Ramezani et al., 2014; Weathers et al., 2016; Yeşilçınar et al., 2018). This concept has been expanded beyond religiosity in the 1960s and 1970s (Moberg, 2012, p. 17). Spirituality is associated with religiosity but differs from it (Murgia et al., 2020; Soósová et al., 2021) by being a broader concept (Berry, 2005). This difference, requiring conceptual clarity, is not fully understood (Berry, 2005; Gall et al., 2011; Buck, 2006; Soósová et al., 2021). In this research, spirituality is conceptualised broadly (Gall et al., 2011) and religion is seen as one aspect of many.

Research data on the spirituality of older people living with dementia has increased recently (Kevern & Stifoss‐Hanssen, 2020; Palmer et al., 2020). Older people are informants in very few of these studies (e.g. Carr et al., 2011; Chen et al., 2019; Kirkland et al., 2014; Wu & Koo, 2016) and are often excluded from research because of communication challenges (Johnston et al., 2016). However, older people's understanding of their spiritual position is essential to nurses supporting a person's spirituality (Missel & Birkelund, 2020). Similarly, close family and friends are often entwined in the lives of these older people (Gibson et al., 2021) and so are useful research informants in this type of study.

## 2 MATERIALS AND METHODS

### 2.1 Data collection

We collected the data from September 2017 to March 2020 through interviews (Kvale, 1996). The first author conducted the interviews individually or with a family member. The interviewer had previous experience of conducting interviews and of communicating with older people living with dementia. Prior to the interviews, the researcher reflected her own pre‐understanding of the topic.

The interviews took place in private rooms or the homes of older people living with dementia, to ensure that the interviewees were at ease and could speak freely. The interviewer asked the participants to talk about their experiences of spirituality and support provision in their nursing care. To encourage the participants to continue their story, the interviewer used probing questions. The interviews lasted from 56 to 110 min, were recorded and transcribed verbatim.

### 2.2 Participants

Those older people living with dementia were included in the research if they were: adults 65 years of age or older; able to express themselves verbally; diagnosed with dementia; able to give consent and had a proxy who could confirm the consent; and home care clients or lived in care settings. The family members who took part in the interviews were all familiar with the nursing of older person living with dementia.

We recruited a purposive sample of participants in home care and long‐term care settings in southern Finland. We asked nurses to give potential participants an information sheet about the study. When someone expressed an interest in participation, the first author contacted him/her by telephone to talk more about the study and to make an appointment for an interview. Saturation of the main themes was achieved when altogether 19 participants were interviewed in 10 interview settings. None of the participants asked to withdraw from the study.

Ten of the 19 participants were older people living with mild or moderate dementia and nine others were their family members. The ages of the older people with dementia varied from 76 to 92, three were male and seven female. The older people were diagnosed with different types of dementia. Seven were cared for at home as home care (HC) clients and three were residents in long‐term care (LTC) settings. Of the nine family members, seven were female and two were male. Four of the family members were daughters, three were spouses and two were other types of family members. We asked all participants to consider spirituality support from the perspective of the older people living with dementia and their comments were given equal weighting. One older person living with dementia (participant no. 5) participated without a family member.

Most of the participants were Evangelical Lutherans but religion does not play a significant role in the daily lives of most Finnish people who belong to the church. Some of the participants belonged to other Christian churches or did not belong to any church. The characteristics of the participants are presented in Table 1. We added pseudonyms to the participants post‐hoc.

**TABLE 1:** Characteristics of the participants

Participant no. (pseudonym)	Age	Gender: Man (M) or woman (W)	Older person (OP) or family member (FM)	Family member's relationship to the older person	Diagnosis of the older person	Home care (HC) or long‐term care (LTC)	Spiritual background, if expressed
1 (Mary)	92	W	OP		Alzheimer's	LTC	Adventist
2 (Robert)		M	FM	Other			Evangelical Lutheran
3 (James)	83	M	OP		Alzheimer's	HC	Not disclosed
4 (Linda)	66	W	FM	Spouse			Christian
5 (John)	80	M	OP		Alzheimer's	HC	Irreligious
6 (Bill)	80	M	OP		Alzheimer's	HC	Evangelical Lutheran
7 (Patricia)		W	FM	Spouse			Evangelical Lutheran
8 (Barbara)	76	W	OP		Vascular dementia	HC	Evangelical Lutheran
9 (Carol)	45	W	FM	Daughter			Evangelical Lutheran
10 (Nancy)	92	W	OP		Alzheimer's	HC	Evangelical Lutheran
11 (Sandra)	66	W	FM	Daughter			Not disclosed
12 (Susan)	78	W	OP		Unspecified	HC	Evangelical Lutheran
13 (Rickhard)	80	M	FM	Spouse			Atheist
14 (Betty)	87	W	OP		Alzheimer's	LTC	Lestadian
15 (Shirley)		W	FM	Other			Evangelical Lutheran
16 (Eva)	97	W	OP		Alzheimer's and Lewy Body Dementia	HC	Evangelical Lutheran
17 (Sue)		W	FM	Daughter			Not disclosed
18 (Edith)	89	W	OP		Unspecified	LTC	Evangelical Lutheran
19 (Julie)		W	FM	Daughter			Not disclosed

### 2.3 Interpretation

We managed the collected data as a whole and built the understanding of the spiritual perspectives and the nursing support the participants received for their spirituality, incrementally. We adapted Ricoeur's (1976) theory for hermeneutical interpretation of the data to provide a framework to understand the experiences of the participants. The first author conducted the interpretation in three steps: naïve reading, structural analysis, and comprehensive understanding (Charalambous et al., 2008; Charalambous & Charalambous, 2016).

Naïve reading took place alongside the transcription of the data and required a careful study of the text to formulate the basic structure of the phenomenon. Second, in the structural analysis, we grouped meaning units using the structure outlined in the naïve reading. Ricoeur (1976, p. 1) states that the units of language and thought should include at least a name and a verb so that was the minimum used in this interpretation process. We condensed meaning units into sub‐themes and further into themes (Table 2). When we formulated a theme, we named it and used to search for new meaning units and sub‐themes in relation to it, thus ensuring the meaning of the theme within the data. Third, we constructed a comprehensive understanding of the participants' lived experiences based on the naïve reading and the structural analysis by furthering the interpretation. In formulating the comprehensive understanding, we revisited findings, in the light of the researcher's pre‐understanding and previous knowledge of the phenomenon (Charalambous, 2014). The interpretation process did not proceed in a straight line from step to step but circumferentially which is usual in hermeneutics (Charalambous, 2014; Charalambous et al., 2008; Debesay et al., 2008).

**TABLE 2:** An example of the interpretation

Meaning unit	Condensation	Sub‐theme	Theme
*“I would go to church, but it's so difficult to move around.”*	Going to church	Religion	Supporting the spirituality through elements of spirituality
*“Listen, if there is something difficult and you pray for help with it, it will become easier many times. Of course, not just when I wanted it. But every morning and evening I pray.”*	Praying		
*“There are people nearby who can be trusted and who I can expect to support and to be close.”*	People you can trust	Meaningful relationships	
*“Taking care of others is important and certainly a source of joy and strength for her.”*	Taking care of other people		
*“Nature speaks and the creator speaks through it.”*	Creator speaking through nature	Nature	
*“I feel that there is no need for a church or a congregation, but I can establish my church by the sea or anywhere.”*	Church by the sea		
*“Art, making art, and art in general can also be spiritual.”*	Spirituality in art	Art	
*“Spirituality is the sensations that grow from music.”*	Spirituality in music		

### 2.4 Ethical considerations

We conducted this research according to the National Law in Finland and in accordance with the Declaration of Helsinki. The first author informed potential participants about the study both orally and in writing. They were reassured that: their participation was voluntary; they could withdraw from the study at any time; the data would be anonymized; and withdrawal would not in any way impact their care and services. We used pseudonyms to ensure participants' privacy. Each participant signed a consent form. Older people living with dementia can be considered vulnerable because it is not always possible to assess their ability to give informed consent (Racine & Bracken, 2019). Therefore, family members confirmed the consent of the older people living with dementia to whom they were close.

During the interviews, we paid particular attention to make sure that the older people with dementia felt at ease by conducting the interviews in an environment familiar to them and with a person close to them if they so wished. Ethical approval was given by the university committee of ethics. After that, permissions to conduct the study were received from each organisation.

## 3 RESULTS

### 3.1 Naïve reading

The naïve reading revealed that the personal meaning given to spirituality, created a background which could be used to support the spirituality of older people living with dementia. This support consisted of addressing and meeting spiritual needs individually using different elements of spirituality. The results showed that participants living with dementia needed spiritual support to maintain meaning and purpose in their lives. The participants also outlined some challenges to the spiritual support in their nursing care.

### 3.2 Structural analysis

In the structural analysis, we identified five themes: Personal meaning of spirituality as a basis for supporting spirituality; addressing the individual spiritual needs of older people living with dementia; meeting their spiritual needs through elements of spirituality; meaning in life through spiritual support; and challenges in supporting spirituality in nursing care.

#### 3.2.1 Theme 1: Personal meaning of spirituality as a basis for supporting spirituality

The participants understood spirituality in many ways. Spirituality was considered something profound, with a deeper meaning in humans. It was considered a resource of life.

Spirituality is humanity; part of being human. (2 Robert)

Some participants felt that they were spiritual without being religious. Others did not identify a difference between spirituality and religiosity. The participants highlighted the importance of individuality in supporting spirituality. Participants emphasised the fact that they believe spirituality to be a highly personal matter that can be affected by the unique experiences and perspectives of a person. One older person with dementia expressed this as:

Spirituality is my way of travelling (1 Mary)

#### 3.2.2 Theme 2: Addressing individual spiritual needs

The participants thought that spirituality should be embraced within nursing care and could not be ignored. The older people living with dementia experienced emotional pain and anxiety because of memory loss. For example, some participants felt that their dementia had caused social distancing from their family and friends.

…everyone has spiritual needs and that cannot be ignored. Of course, on the terms of each person. (2 Robert)

Indeed, this dementia has been one thing that has distanced her a little from the herd, her own family.(15 Shirley)

The participants believed there were different ways to address the spiritual needs of older people living with dementia. This was thought necessary because people with advanced dementia would not be able to express themselves verbally. In these cases, the participants believed it was essential to collect the person's spirituality information using alternative methods.

There were questions in the nursing home's information form about her spiritual background. (2 Robert)

#### 3.2.3 Theme 3: Meeting the spiritual needs of older people living with dementia through elements of spirituality

The participants outlined four different ways through which spirituality could be supported: religion, meaningful relationships, nature, and art.

##### Supporting spirituality through religion

The participants outlined several ways that the spirituality of older people living with dementia was supported through religion: spiritual literature; parish activities; spiritual radio and TV programs; spiritual music; and praying. Overall, it was important to the participants that nurses respected the religion of older people living with dementia.

Spiritual literature, such as the Bible or other spiritual books, was important to the participants. Some older people living with dementia were still able to read while others needed someone else to read to them.

I read the Bible. (1 Mary)

Some participants took part in parish activities such as religious services, in nursing homes or in churches, which supported their spirituality. Though challenging at times, some participants felt that the atmosphere in a church was better for spiritual support.

I would go to church, but it's so difficult to move around.(1 Mary)

Church services where the special needs of people with dementia were taken into consideration, were found to be easier to participate in and, making it even easier, Chaplain's visits were appreciated. As a part of the church service, Holy Communion was perceived as important for some participants, and socially, many wanted to discuss their memories of participating in different church activities when they were younger.

Spiritual radio and TV programs became important for many participants as their physical ability decreased. Some family members helped the older person and their nurses by pre‐selecting a spiritual radio channel.

The Christian radio channel is pre‐selected on that radio if she wants to listen to that.(2 Robert)

The participants felt that an easy way to support their spirituality was to be able to switch on the radio or television at the right time, on the right channel so that they could listen to a religious broadcast. Some participants listened to recordings of poems, Bible verses and spiritual music.

Spiritual music was considered a useful way of supporting spirituality. Many older people living with dementia had their own hymnals and vocal groups sometimes visited the nursing homes. In addition to listening to the music some also enjoyed singing spiritual music, recalling the lyrics of songs even in advanced dementia.

They [older people living with dementia] were there [in nursing home], sleeping with their mouths open, and then when spiritual music was sung, they would immediately begin to move their mouths. (7 Patricia)

The participants prayed. Some used regular and frequent prayers, for example, every night, every morning, or before the meals whilst others prayed only in difficult life situations. Some prayed in their own words while others used familiar prayers they remembered. Some participants wanted to say a prayer in the interview and others used intercession as a way of helping other people when their physical ability had decreased. The participants believed that prayer made difficult things easier and helped them to relax.

Listen, if there is something difficult and you pray for help with it, it will become easier many times. Of course, not just when you wanted it. But every morning and evening I pray. (1 Mary)

Faith seemed to remain even when cognition decreased. This included faith in God, trust in God's guidance and continued belief in the afterlife.

Faith and trust, hope for the future. These remain. (3 James)

##### Supporting spirituality through meaningful relationships

Some participants felt that the spirituality of older people living with dementia can be supported through meaningful relationships with the family and other people. Life was thought to be empty without other people. It was considered spiritually supportive to have people nearby who an older person living with dementia could trust and who was able to provide support and comfort.

There are people nearby who can be trusted and who I can expect to support and to be close.(8 Barbara)

The participants considered their personal roots and home region the basis for their spirituality, and their family a source of joy and strength. This occurred mostly when family members, including children and grandchildren, took care of each other, accepting the older person and the effects of their dementia. In its simplest form, the support of spirituality from nurses could occur when someone asked how they were in a moment of need.

When your own child is born, there is a moment in it that you feel spiritual, that it is somehow sacred.(5 John)

It was such an empathetic gesture when he [nurse] came to ask how I was. (8 Barbara)

Some participants believed that a meaningful interpersonal relationship supporting spirituality, may also emerge between an older person with dementia and the nurse. This required the nurse to have suitable social values which was evident in the respect shown for the people with dementia in their way of working. When the older people living with dementia felt that the nurses cared about them, they were considered as persons, not just as patients, and accepted as they were.

It's about the love we should show to other people. It becomes visible and is experienced in that nursing work. (15 Shirley)

It was easier for the participants to talk about spirituality with nurses they knew well. If the nurses were changed frequently, deeper discussions became more difficult. Discussion about spiritual matters need an open relationship with an understanding nurse ready to listen. Confidentiality was considered essential in the nursing support of spirituality. The participants enjoyed the company of nurses who were able to be present, and their presence was relaxing. For spiritual support, nurses needed time to stay by their side and comfort them at bad times.

It is to have courage to be present. It can be easy for the nurse if she dares to be present and open to listen and to be there for another person. (15 Shirley)

##### Supporting spirituality through nature

The participants considered nature a relaxing spiritual element. They talked about their spiritual experiences by the sea, in the garden, in the woods, in the park or at the summer cottage. They described the roar of the waves, the sound of the leaves on the trees in the wind, flowers, birds, and squirrels. Nature was considered a *“health battery”*_*6*_ and *“charging station”*_*11*_. Some participants believed that they did not need churches because they had their own church in nature.

Nature speaks and the Creator speaks through it. (9 Carol)

I feel that there is no need for a church or a congregation, but I can establish my church by the sea or anywhere. (9 Carol)

##### Supporting spirituality through art

Some participants experienced spirituality support through art. Art was experienced as healing the whole person, touching something deep in us. This was what the participants experienced made art spiritual. This included for example music, poetry, visual arts, and handicrafts. Playing instruments had become more difficult for some participants, but listening to music was important for many.

Spirituality is the sensations that grow from music.(5 John)

Some of the older people living with dementia had written poems themselves and wanted to read them aloud in the interview. Some had found other touching poems they wanted to recite. The participants wanted to show paintings or handcrafts they felt were supporting their spirituality.

Art, making art, and art in general can also be spiritual. (3 James)

#### 3.2.4 Theme 4: Meaning in life through supporting spirituality

The participants reflected that supporting spirituality helped older people with dementia to maintain meaning in their life, made them happier and gave them joy. Spirituality also gave hope for the future and reduced fear, helping them to prepare for death whilst still feeling safe and maintaining faith in life. Spiritual support, and their interest in spirituality seemed to help the participants to cope with dementia by increasing calmness, providing some inner peace and confidence.

Faith creates safety. It is the crystallization of what spirituality can be for either him or anyone. (4 Linda)

When adversity comes, I believe in God and all that is good. That brings a feeling of calm so that there is no such restlessness. Spirituality is such that there is no fear. (10 Nancy)

#### 3.2.5 Theme 5: Challenges in the support of the spirituality of older people living with dementia in nursing care

The older people living with dementia did not seem to want to bother busy nurses or parish workers with their spiritual needs as they felt that the nurses and parish workers did not have enough time. They also thought that not all the nurses understood how important spirituality could be for some older people living with dementia and that some did not have the competence to support spirituality.

Well, they [nurses] have so many people to see to that they are in a hurry with that. (14 Betty)

## 4 DISCUSSION

### 4.1 Comprehensive understanding and reflections

The aim of this research was to understand comprehensively, how older people living with dementia and their family members experience spirituality and its support in nursing care. To achieve this goal, the researchers worked between the study data, previous literature, and the researcher's pre‐understanding of spirituality, moving alternately from the whole to individual parts and back to the whole as a hermeneutic circle (Charalambous, 2014). The researchers combined the information from the different sources, in Ricoeur's (Ricoeur, 1976) words, into a *being‐in‐the‐world* conception of the phenomenon. It was through this lens that a personal sense and comprehensive understanding of the data was derived.

The complexity of defining spirituality has been widely recognised (Berry, 2005; Connolly & Moss, 2021). This is indicated by the large number of concept analyses (e.g. Buck, 2006; Il et al., 2017; Murgia et al., 2020; Weathers et al., 2016; Yeşilçınar et al., 2018) on the subject. In this research, the participants' personal understanding of spirituality created the framework for the experiences of spiritual support. There were many different interpretations of spirituality within the participant group, even though their cultural background was homogenous. In most cases, the participants did not equate spirituality with religion, but with the experience of the meaning of life. This supports Murgia et al., 2020 who in their concept analysis stated that though it is common to combine spirituality and religion, the meaning or purpose of life has also been associated with the concept of spirituality Murgia et al. (2020).

It has been noted that even in secular societies, people have spiritual needs (Timmins et al., 2016). The participants in this study concurred with this view but the content of the needs varied. Dementia was seen as a severe disease that, at times, caused emotive anxiety and feelings of loss. Many participants were moved to tears during the interviews when talking about spirituality. We considered this an indication that spirituality was perceived as profoundly emotional for the participants. The participants felt that support with their spirituality could help to deal with these feelings, improving their ability to cope. The perspective of coping with dementia in spiritual needs has previously been highlighted (Chen et al., 2019).

We found four main elements of spirituality to describe the participants experiences of spirituality. The elements were religion, meaningful relationships, nature, and art. All these aspects of spirituality have been observed previously (Carr et al., 2011; MacKinlay & Trevitt, 2010). In the light of this knowledge, these elements can be utilised in nursing to support the spirituality of older people living with dementia.

Religion has been seen as an important aspect of spiritual support in earlier studies (e.g. Carr et al., 2011; Chen et al., 2019). In relationships, love and its depth, as well as life itself, was considered spiritual. Chen et al. (2019) found that connectedness with other people was a particularly important element of spirituality for non‐religious older people living with dementia and this was supported in this current study. In previous research, Soósová et al. (2021) reported that connectedness within the self was also linked to spirituality. This self‐connectedness was not demonstrated in this study which may be concerned with the self‐fragmentation of dementia. The participants may have not been able to look within themselves and made more use of external elements to support their spirituality.

Experiencing spirituality through nature required real or virtual nature experiences. The connection between nature and spirituality has emerged in previous studies (Goodall, 2009; Soósová et al., 2021). In this study participants repeatedly emphasised the importance of nature in their spiritual experiences.

Through art, spirituality was experienced, for example, through a meaningful poem but most often it was music. This finding supports Connolly and Moss (2021) who reported older people living with dementia expressing their spirituality through music.

According to this study, to support the spirituality of older people living with dementia, an understanding of which elements are central to each person's spiritual experience is required. Additionally, in this study, the spiritual support through multiple senses deepened the experience of older people living with dementia. For example, in religion, the Holy Communion touched different senses simultaneously. Similarly, in nature, the combination of aromas, sounds, and tastes took the older people living with dementia back to a spiritual experience that was familiar to them. Television and radio have been used to support spirituality for a long time, but the use of various live streams and virtual experiences has also increased. These avenues of support may be more important when real life contacts are minimised, for example during the recent Cov‐Sars‐2 pandemic.

In this study, meeting the spiritual needs of older people living with dementia helped to give their life meaning and purpose. The participants believed that a wide variety of experiences, occurrences and items were the components of their spiritual lives and so for them had spiritual meaning. For example, handicrafts made by themselves or by loved ones, the landscape seen from their home window, or a picture of a grandchild could be considered spiritual. This variation has been seen as a challenge in spirituality research (Berry, 2005). In this research, the sense of spirituality arose from the meaning in life that these components of spirituality symbolised. This finding was also highlighted by Carr et al. (2011) and Zibad et al. (2017).

In the interviews, the participants listed those components of their life that supported their spirituality. The lists were often followed by an additional sentence explaining that the components listed were spiritual in a personal sense and included: *“But this is not for everyone”*, *“For me, this is important”, “But in a personal way, of course.”*

Supporting spirituality was thought to be an essential part of holistic nursing care. However, the participants acknowledged that meeting spiritual needs in nursing care could be challenging. These findings are consistent with previous research (MacKinlay, 2008). This challenge is considered to be related to the complexity of spirituality as a phenomenon (Connolly & Moss, 2021). In this research, the time and competence resources of the nursing staff were thought to be the most important challenges. Some nurses were considered to have not enough knowledge of spiritual needs or a sufficient understanding of the spiritual dimensions of a person. The participants also felt that nurses did not always have enough time to provide spiritual support. Despite the challenges related to time, other resources and the competence of nurses, the participants did have experiences with nurses who supported their personal sense of spirituality in the way they worked. This included the ability to be present and know the person.

Supporting the spirituality of older people living with dementia involved some of the same elements as supporting the spirituality of other groups. Individuality and subjectivity as principles of supporting spirituality have already been highlighted in previous studies (e.g. Berry, 2005; Connolly & Moss, 2021; Daly et al., 2019; Zibad et al., 2017) and were confirmed in the results of this study. However, as dementia progresses, the older people living with dementia are at the particular risk of failing to have their spiritual needs met because of an impaired ability to express themselves, as also noted by Daly et al. (2019). Older people living with dementia may not be able to care for their spirituality on their own but need the external help of a knowledgeable, experienced and understanding person who has a presence and the time to provide this support. This is a challenge for nurses which has increased during the Cov‐Sars‐2 pandemic, because of social distancing and quarantine regulations (Bolt et al., 2021).

In this study, common to all experiences of spirituality, support is often experienced as a presence, as also observed by Carr et al. (2011). Spiritual support requires understanding between two people which may only be a moment, between the two, out of the hectic world.

### 4.2 Methodological considerations and limitations

Spirituality as a concept and as a subject of research is constantly evolving. We conducted qualitative research to bring more depth to the understanding of the phenomenon. Recruitment was quite challenging as usual in studies having people with dementia as informants (Juaristi & Dening, 2016; Tam et al., 2021). Recruitment was made more challenging as the goal was to find pairs of participants; an older person with dementia and a family member, both interested in participating in the study. The subject of the study, being abstract and intangible, may have prevented some from participating. However, the participants were able to discuss the topic to a level that facilitated the research.

Transferability was increased by describing the participants and context as accurately as possible and by using purposeful sampling (Cypress, 2017). The sample size was sufficient for the researcher to gain a comprehensive understanding of the phenomenon using an adaptation of Ricoeur's theory of interpretation. However, the sample was small, and the cultural background of the participants was homogenous. This makes it difficult to generalise the findings.

We used quotes from the interviews in the interpretation to add transparency and credibility. It should be noted that the participants spoke Finnish, so the quotes have been translated from Finnish to English. The methods of data collection did not pose any significant difficulties for the participants. Ricoeur's hermeneutics was a useful method for understanding multidimensional data leading to the core matter.

## 5 CONCLUSIONS

The study findings using an adaptation of Ricoeur's interpretation theory indicate that supporting the spirituality of older people living with dementia in nursing is based on the older people's personal view of spirituality. Caregivers should identify these personal views to support people with spiritual needs which may increase over time as the dementia progresses. This study reports that the spirituality of these older people can be supported through religion, meaningful relationships, nature, and art. The methods of support will depend on how each person expresses their spirituality, and through which media they choose their expression at the time. Cultural and religious diversity of society has been increasing which needs to be considered when managing spiritual support. The time taken to do this will be useful as it is one way to help a person to maintain meaning and purpose in life and engender a feeling of security in the face of serious illness.

The limited time and competence of the nurses discussed during the study is a challenge to the realisation of useful spirituality support. Nurses need more education to be able to learn and develop experience in supporting the spirituality of older people living with dementia in practice. At the strategic level, the overall well‐being of older people living with dementia should be considered within the holistic organisation of care, alongside physical and mental health care. Organisational support is needed for nurses to integrate spiritual care into practice. More research is needed to deepen the understanding of the phenomenon in different cultures and micro‐cultures.

## 6 IMPLIATIONS FOR PRACTICE

Our findings suggest that supporting spirituality is included as part of nursing care for older people in home care and long‐term care settings. We suggest that supporting spirituality is implemented individually, respecting the way in which each older person living with dementia expresses their spirituality and through which elements it manifests. In the sustainable development of nursing care for older people with dementia, it is essential to hear the views of people with dementia. The findings of this research can be used in the training of nurses so that they develop the competence and experience to support the spirituality of older people living with dementia.

## FUNDING INFORMATION

This study was supported by funding via The Betania Foundation and The Finnish Nursing Education Foundation sr.

## CONFLICT OF INTEREST

The authors declare that they have no known competing financial interests or personal relationships that could have appeared to influence the work reported in this paper.

## Data Availability

The data that support the findings of this study are available on request from the corresponding author. The data are not publicly available due to privacy or ethical restrictions.
